# Equine castration in Sweden: a retrospective study of postoperative complications across field and hospital practices

**DOI:** 10.1186/s13028-026-00889-y

**Published:** 2026-11-13

**Authors:** Ida Sjöberg, Johan Lundgren, Kristin Syrén

**Affiliations:** Department of Clinical Sciences, Faculty of Veterinary Medicine and Animal Science, Swedish University of Agricultural Science (SLU), Box 7054, Uppsala, S-750 07 Sweden

**Keywords:** Antibiotics, Horse, Orchidectomy, Surgical site infection

## Abstract

**Background:**

This retrospective study describes and compares the surgical techniques and medical protocols used for castration of male horses in Swedish ambulatory practices (Field group) and in Swedish equine hospitals (Hospital group). Data on patient characteristics, perioperative medical protocols, surgical techniques and postoperative complications were collected as documented in patient records of both groups. Descriptive statistics, univariable and multivariable analyses were used to evaluate factors associated with postoperative complication.

**Results:**

The overall complication rate was 35% in the Field group and 13% in the Hospital group. This difference remained significant after adjusting for age and breed in a multivariable analysis (OR 4.5; CI 3.18–6.36). In the Field group 511/521 horses (98%) were castrated standing using sedation and local analgesia, incisions were left un-sutured in all horses (100%). The surgical technique was described in 230/521 records (44%), the open technique was used in 194/230 horses (84%) and the half-closed technique was used in 36/230 horses (16%). Complication and surgical site infection were associated with higher odds in horses that did not receive perioperative antimicrobial prophylaxis (OR 1.5; CI 1.01–2.36 and OR 1.7; CI 1.08–2.82 respectively). In the Hospital group, 554 stallions were castrated under general anaesthesia, and the incisions were sutured in 473/554 horses (85%). The closed technique was used in 503/554 horses (91%) and the half-closed was used in 51/554 (9%). Perioperative antimicrobial prophylaxis was administered to all horses in the Hospital group. Postoperative antimicrobial prophylaxis did not decrease the odds of surgical site infection in any of the groups.

**Conclusions:**

In Sweden, standing, open castration with un-sutured incisions was preferred in ambulatory practice, whereas a closed, sutured technique dominated in the hospitals. Field castration was associated with a substantially higher odds of postoperative complications, compared with hospital-based castrations performed under general anaesthesia. These findings provide locally relevant evidence to guide clinical decision-making and antimicrobial stewardship and may contribute to the ethical discussion regarding the use of standing castration when alternatives are feasible.

**Supplementary Information:**

The online version contains supplementary material available at https://doi.org/10.1186/s13028-026-00889-y.

## Background

Castration of the male horse is the most frequently performed surgical procedure in horses, and castration techniques as well as anaesthetic protocols have been well described in the literature [1–6], however not specifically for Sweden. Castration techniques are grossly separated in field castrations and recumbent anaesthetic procedures performed in a surgical theatre. Under field conditions, the skin incisions are generally left un-sutured, whereas castration under general anaesthesia permits an aseptic technique and primary closure of the incisions. Complication rates vary widely, depending on surgical technique, perioperative conditions and the definition of complication, which confounds comparisons across studies. Nevertheless, although standing castrations are an elective surgery, they have consistently been associated with higher complication rates ranging from 10% to 60% [2, 6, 7] than other elective procedures with reported rates of 17% [8].

The most clinically relevant complications after equine castration include haemorrhage, herniation/eventration, and surgical site infection (SSI). Haemorrhage and herniation are largely structural, technique-dependent events linked to tissue handling and haemostatic strategy and are typically apparent perioperatively or shortly thereafter. In contrast, the SSI risk is more strongly influenced by asepsis, environmental contamination, and postoperative management. Early diagnosis can be challenging as some degree of swelling and wound exudation may be expected and are subjectively assessed. Consequently, SSI definitions and follow-up markedly affect reported incidence [9].

The reported incidences of SSI in un-sutured castrations range from 2% to 30% [2, 10, 11], and SSI rates after sutured castrations under general anaesthesia ranges from 0% to 10% [2, 12, 13]. Since few studies have reported SSI rates after sutured castration performed without antimicrobial prophylaxis [14, 15], quantifying complication rates by castration setting and closure approach in Sweden is warranted to inform evidence-based prophylaxis policies and antimicrobial stewardship.

This retrospective chart review describes surgical techniques and perioperative medical protocols used for castration of horses in Swedish ambulatory and hospital practice. Postoperative complication rates and associated factors, with particular emphasis on SSI, were investigated and compared between these two types of equine practice.

## Methods

### Medical records

Medical records of colts and stallions castrated under ‘field’ conditions during 2019 were obtained from the District Veterinary Organisation (DVO), a state-governed nationwide ambulatory veterinary service (Field group). In addition, records from castrations performed under general anaesthesia were acquired from three equine hospitals: A, B and C, between 2014 and 2020 (Hospital group). Due to the retrospective review of medical records only, institutional ethical review was not deemed necessary.

### Data collection and inclusion criteria

Records of castrated horses with two descended testes in the scrotum were included in the study. Horses with urogenital pathology, castrations with an inguinal approach, or records lacking sufficient data were excluded. Collected data included age, breed, perioperative and postoperative medication, anaesthetic protocol, surgical technique (i.e. open, closed, or half-closed technique) [16], use of a ligature around the spermatic cord, and wound closure (i.e., sutured or un-sutured). The month in which the surgery was performed was registered and categorised into one of four meteorological seasons. Any notation of a postoperative complication in the medical record up to 6 weeks after castration was categorised according to the following definitions:

#### Non-infectious postoperative complication (non- SSI)

Excessive scrotal swelling without fever (rectal temperature ≤ 38.4 °C), consistent with non-infectious causes such as haemorrhage, seroma, haematoma, and/or prolapse of tissue (e.g., subcutaneous tissue) or evisceration, in the absence of purulent discharge or devitalised infected tissue.

#### Surgical site infection (SSI)

Evidence of infection was defined as any of the following: excessive scrotal swelling with fever (rectal temperature > 38.4 ℃); and/or purulent drainage from the incision; and/or devitalised and infected tissue at repeated surgery.

Because this was a retrospective study, SSI could not be confirmed by standardized clinical scoring or cytology; therefore, SSI was defined using objective systemic findings (fever) and/or gross local evidence of infection (purulence or devitalised infected tissue), rather than bacterial culture. When rectal temperature was not recorded, classification relied on documented purulent discharge and/or devitalised infected tissue.

#### Anaesthesia

Any catastrophic event during anaesthesia or recovery, i.e., cardiac arrest, fracture or myopathy. Fever within 48 h post-surgery without any clinical signs at the surgical site, or signs of respiratory disease or colic within 2 weeks post-surgery.

### Castration techniques

The terms open, closed or half-closed technique in this study are used according to previously established definitions [16]. Briefly, open technique refers to when each testis is exteriorised via an incision through the parietal tunics. After crushing the distal portion of the spermatic cord with an emasculator the testis is removed, leaving the parietal tunic and the cremaster muscle in the horse. In the closed technique, the parietal tunic is not incised and the cord, including the cremaster muscle is crushed with the emasculator, and the testis and part of the parietal tunic are excised. The half-closed technique is a combination of the two techniques, where the parietal tunic is incised to allow inspection of its content before the cord, covered by the parietal tunic, is crushed and the distal part removed. Regardless of the used technique, the spermatic cord can be ligated, and the skin incisions can be sutured or left un-sutured.

### Statistical analysis

Collected data was analysed using descriptive statistics. Since most castrated horses represented younger individuals, age was not normally distributed and therefore categorised as ≤ 2, 3–4, and ≥ 5 years for the statistical analyses. Breeds with relatively few individuals were combined into a single category.

Associations between explanatory variables and postoperative complication (Yes/No) or SSI (Yes/No) were analysed in the full study population and separately within the Field and Hospital groups using univariable chi-square tests and multivariable logistic regression models. In the full study population, group (i.e. Field or Hospital), age and breed were included in the multivariable model based on their biological relevance as potential confounders. A threshold of *P* < 0.2 in the univariable analysis was used to select variables to be entered into separate multivariable models for the Field and Hospital groups and age and breed were forced into all models. Variables were removed using stepwise backward selection until only significant variables remained. Statistical significance was set at *P* < 0.05. All analyses were conducted using the statistical software JMP Pro16 (SAS Institute, NC, USA).

The results from the univariable analyses are presented in Additional file 1. Odds ratios for variables retained in the multivariable models for complication and SSI are presented in detail in Additional Files 2–4.

## Results

### Medical records

Records of 521 and 554 castrations were reviewed in the Field and Hospital groups, respectively. The full study population consisted of Standardbred trotters, warmblood horses, ponies, cold-blooded horses, Icelandic horses, and other miscellaneous breeds (Table 1). The median age in the Field group was 2 years (range < 1–12 years), and 3 years (range < 1–19 years) in the Hospital group. Age was missing in 88 records in the Field group. Horses were castrated all year round in both groups, but a majority, 368/521 (Field) and 338/554 (Hospital), were castrated during spring and autumn.

**Table 1:** The distribution of age and breed in the full study population and in the two groups

Breed (*n*)	Field *n* (% of *N*)	Hospital *n* (% of *N*)
Cold-blooded horses (82)	71 (13.6)	11 (2.0)
Icelandic horses (76)	46 (8.8)	30 (5.4)
Other (158)	127 (24.4)	31 (5.6)
Ponies (119)	105 (20.2)	14 (2.5)
STB (331)	28 (5.4)	303 (54.7)
WBL (309)	144 (27.6)	165 (29.8)
**Total (N)**	**521**	**554**
**Age (n)**		
≤ 2 (767)	315 (72.7)	252 (45.5)
3–4 (311)	104 (24.0)	207 (37.4)
≥ 5 (109)	14 (3.2)	95 (17.1)
**Total (N)**	**433** ^**#**^	**554**

# Missing values: 88

Values have been presented as numbers with respective percentage of total population in brackets. Percentages were calculated within each column

Abbreviations: STB; Standardbred trotter, WBL; warmblood horse

From the medical records in the Field group the following information was revealed: Perioperatively, 491 horses (94%) were administered NSAIDs, benzylpenicillin was used in 233 horses (45%), and two horses received trimethoprim sulfadiazine (0.4%). Postoperatively, 376 patients (72%) were prescribed NSAIDs, and 86 (17%) continued with an extended course of prophylactic benzylpenicillin or trimethoprim sulfadiazine. In 511 horses, the castrations were performed under standing sedation (98%), using a protocol that included a combination of an alpha-2 agonist and an opioid, with or without acepromazine. Ten horses were castrated recumbent (2%) under dissociative anaesthesia with ketamine added to the protocol. Surgical technique was described in 230 of 521 records (44%) (Table 2). The open technique was used in 194 horses, whereas the half-closed technique was used in 36 horses. Ligatures were used in three cases, and all skin incisions were left un-sutured to heal by second intention. Discharge instructions were given to all patients, informing the owners to contact the veterinarian if any complication occurred.

From the medical records in the Hospital group the following information was revealed: All patients received intravenous NSAIDs (flunixin meglumine 1.1 mg/kg or meloxicam 0.6 mg/kg), and benzylpenicillin (9.5–19 mg/kg) before induction of anaesthesia. Postoperatively, 308 horses (56%) were treated with NSAIDs, and 240 (43%) continued with an extended course of prophylactic benzylpenicillin or trimethoprim sulfadiazine. Routine anaesthetic protocols were similar across hospitals, using acepromazine, alpha-2 agonists, and opioids as pre-medications, followed by induction with benzodiazepines and ketamine. Anaesthesia was maintained using isoflurane. The preferred castration technique varied between surgeons (*n* = 13). The closed technique was used in 503 horses, and the skin closed with primary suture in 473 horses (Table 2). In all castrations the spermatic cord was crushed using an emasculator and the vaginal cavity was sealed using a compressive ligature. Three surgeons used the half-closed technique (9% of the castrations), and five surgeons sometimes left the skin to heal by second intention (15% of the castrations). Horses were normally discharged from the hospitals the same day or the day after the castration, with instructions to contact the hospital if any complication occurred.

**Table 2:** Surgical techniques in field and hospital settings and across hospital A–C

Surgical Technique	Field^#^ *n* (%)	Hospital *n* (%)	A *n* (%)	B *n* (%)	C *n* (%)
Open	194 (84)	0 (0)	0 (0)	0 (0)	0 (0)
Closed	0	503 (91)	288 (100)	157 (76)	58 (97)
Half-closed	36 (16)	51 (9)	0 (0)	49 (24)	2 (3)
Primary suturing	0 (0)	473 (85)	218 (76)	205 (99)	50 (83)

# Missing values: 291

Values have been presented as numbers with percentage in brackets. Percentages were based on the total number of individuals in each group (N). Field (*N* = 230), Hospital (*N* = 554); Hospital subgroups: A (*n* = 288), B (*n* = 206), C (*n* = 60)

### Postoperative complications

The overall complication rate was 35% in the Field group and 13% in the Hospital group and the SSI rate was 26% and 4.5% respectively (Table 3).

In the Field group, complications in 13 horses could not be treated successfully by the local veterinarian and were referred to an equine hospital. Seven of these complications were due to postoperative haemorrhage and six because of SSI. In the Hospital group, nine horses required a second surgery, one of these suffered from a seroma formation and the others were diagnosed with SSI. One of these horses was euthanized because of an abdominal abscess in the inguinal region, which did not respond to treatment. Evisceration did not occur in either group, and no catastrophic events were reported during anaesthesia or recovery. Complications in the anaesthesia category consisted of three horses in the Field group and six in the Hospital group that were reported to have fever the day after surgery, and ten horses were presented with colic within 2 weeks of surgery, all in the Hospital group (Table 3).

**Table 3:** Postoperative complications in field and hospital settings and across hospital A–C

Complication	Field *n* (%)	Hospital *n* (%)	A *n* (%)	B *n* (%)	C *n* (%)
SSI	133 (26)	25 (5)	14 (5)	8 (4)	3 (5)
Non-infectious	46 (9)	32 (6)	18 (6)	14 (7)	0 (0)
Anaesthesia	3(1)	16 (3)	4 (1)	12 (6)	0 (0)
Total	182 (35)	73 (13)	36 (13)	34 (17)	3 (5)

Values have been presented as numbers with percentage in brackets. Percentages were based on the total number of individuals in each group (N). Field (*N* = 521), Hospital (*N* = 554); Hospital subgroups: A (*n* = 288), B (*n* = 206), C (*n* = 60)

Abbreviations: SSI; surgical site infection

### Factors associated with complications and surgical site infection

The odds of complications and SSI were higher in the Field group than in the Hospital group after adjusting for age and breed in the multivariable model (OR 4.5; CI 3.18–6.36 and OR 5.9; CI 3.43–9.99 respectively) (Additional File 2). Horses aged ≥ 5 years had higher odds of complication than horses aged ≤ 2 (OR 2.5; CI 1.50–4.24) and all breeds except Icelandic horses had lower odds of SSI than the coldblooded horses (OR 0.3–0.5; CI 0.16–0.92).

In the Field group, complications and SSI were more likely to occur in horses that did not receive antimicrobial prophylaxis perioperatively (OR 1.5; CI 1.01–2.36 and OR 1.7; CI 1.08–2.82 respectively), and in horses that were prescribed NSAIDs postoperatively (OR 2.1; CI 1.36–3.27 and OR 2.5; CI 1.47–4.11 respectively) (Additional File 3).

In the Hospital group, castration of younger stallions was associated with lower odds of complications compared with stallions aged ≥ 5 years (OR 0.3; CI 0.17–0.63 for age ≤ 2 and OR 0.4; CI 0.23–0.83 for age 3–4) (Additional File 4). Surgical site infection was associated with increased odds during spring and autumn compared with winter season (OR 6; CI 1.1–109.8 and OR 10; CI 1.9–185.3 respectively).

## Discussion

The study revealed that castrations in the field were predominantly performed using an open technique on standing sedated horses and the wounds were left un-sutured. In the three equine hospitals, horses were primarily castrated using a closed technique with skin incisions sutured. Field castrations were associated with fivefold higher odds of overall complications and sixfold higher odds of SSI compared with castrations performed in a hospital setting. These findings are consistent with previous reports [2, 17, 18] and confirm that standing castration is associated with a higher risk of postoperative complications than recumbent castrations.

The only source for SSI in castration of a clinically sound horse will be contamination. This can be more easily controlled in a clean surgical procedure than under field conditions. Furthermore, contamination is enhanced by leaving the wounds open for drainage [4]. This retrospective study did not provide infection and inflammatory markers, which prevents establishing an SSI classification. However, the clinical signs documented and the significantly reducing effect of perioperative antimicrobials, as concluded from this study, are strong indications. SSI were reduced, but not completely prevented by perioperative antimicrobials, possibly due to postoperative contamination and/or compromised drainage.

In the present study, a single dose of perioperative antimicrobials was administered to only one third of the horses castrated in the field, but those horses had significantly lower odds of SSI. Perioperative antimicrobials are recommended for surgical procedures in which the expected risk of SSI exceeds 5% in the absence of antimicrobial prophylaxis [19, 20]. However, the use of antimicrobial prophylaxis in un-sutured field castrations remains controversial. Although beneficial effects on the postoperative inflammatory response have been reported [1, 3, 4], these findings do not justify the use of antimicrobial prophylaxis as a substitute for appropriate hygiene and sound surgical technique for preventing contamination.

Perioperative antimicrobials as a standard protocol for castration in the three observed hospitals does not align with the Swedish policy on antibiotic use [21]. During the period this study was performed, published literature describing sutured castrations without antimicrobial prophylaxis was limited [22, 23]. However, it is indicated that the use of a clean surgical procedure for castration does not require routine perioperative antimicrobials [14, 15]. Consistent with the findings in this study, prophylactic antimicrobial use beyond the perioperative period is not useful [24, 25].

Postoperative administration of NSAIDs was associated with a higher incidence of complications including SSI in the Field group. The reported evidence on NSAIDs interference with wound healing are conflicting [26, 27]. The present findings may reflect confounding by indication and/or limitations inherent to the retrospective medical records and should therefore be interpreted with caution pending further studies.

Horses older than five years had higher odds of complication in both the full study population and Hospital group. The association was not retained in the multivariable model for the Field group, possibly due to small number of horses in this age category. Increased age has previously been identified as a risk factor for intra- and postoperative haemorrhage [12, 17, 28]. Although haemorrhage may have contributed to the observed association, this could not be evaluated in the present study.

Postoperative colic was the most common anaesthesia-related complication, occurring exclusively in the Hospital group. Although colic is a known complication of general anaesthesia, with a reported incidence of 2.5–10% after elective surgery [29, 30], it is rarely included in reported complication rates for castrations performed under general anaesthesia [28]. Standing sedation may also be a risk factor for colic [31] but was not observed in the present study. The anaesthesia-related mortality in elective procedures is reported to be low, ranging from 0.6% to 0.9% [31, 32]. This was confirmed in the present study where no catastrophic (i.e. fatal) events occurred during anaesthesia or recovery.

In the study population, no case of evisceration was reported. Evisceration following castration using an open technique is considered an uncommon complication [10, 25]. An apparent exception has been reported in certain breeds, including draft horses and Standardbred trotters, in which incidences up to 8% have been described [33]. In the current study population, few Standardbred trotters were included, and true draught breeds are relatively uncommon in Sweden.

Data for this study were derived exclusively from medical records, which imposes several limitations. Surgical technique was documented in fewer than 50% of the records in the Field group, which may have introduced misclassification bias. However, for standing castrations, the open technique is explicitly recommended in the DVO guidelines, suggesting that the use of this technique was implicit. Peri- and postoperative medications may have been dependent on veterinary surgeon and/or patient, with antimicrobials and/or NSAIDs prescribed preferentially to horses perceived to be at higher risk or showing early signs of complications. Complications may also have been underreported in both groups, as some owners may have sought follow-up care at other veterinary facilities. Standardised follow-up would likely have improved the accuracy of complication estimates; however, given the large number of records retrieved over a six-year period, it was not feasible to contact all owners.

Complication patterns and SSI rates observed in Swedish field and hospital castration were comparable to those reported internationally, suggesting that Swedish practice conditions are not fundamentally different with respect to the main postoperative risks. Importantly, the between-setting differences observed here, with higher odds for complication and SSI after standing, un-sutured field castration compared to sutured castration under general anaesthesia, mirror patterns reported elsewhere [6, 28]. Nevertheless, horse owners often favour field castration for economic and logistical reasons. Standing castrations have been reported to be less costly than castrations performed under general anaesthesia [2], and hospital castration requires transportation, which may be stressful, particularly for young and untrained horses [34]. However, the high SSI-rate observed after standing castration is likely to drive antimicrobial use. Moreover, complications are painful, and veterinarians and horse owners have an ethical obligation to minimise harm to the horse. Accordingly, the routine use of field castration warrants critical evaluation from both a One Health and animal welfare perspective, particularly where sutured castration in a hospital setting is a feasible alternative.

## Conclusions

Standing castration, using an open technique and un-sutured incisions was the predominant approach among ambulatory veterinarians within DVO, whereas a closed sutured technique was preferred in the hospitals. Consistent with previous reports, standing field castration was associated with substantially higher odds of postoperative complications, particularly surgical site infections, compared with hospital-based castrations performed under general anaesthesia. These findings provide locally relevant evidence to guide clinical decision-making and antimicrobial stewardship and may support an ethical discussion regarding the routine use of standing field castration when lower-risk alternatives associated with fewer complications and reduced antimicrobial exposure are feasible.

## Supplementary information

Below is the link to the electronic supplementary material.

Supplementary Material 1

Supplementary Material 2

Supplementary Material 3

Supplementary Material 4

## Data Availability

The datasets used and analysed during the current study are available from the corresponding author on reasonable request.

## Declarations

### Prior publication

This study was based on the Master’s theses of JL and KS. Preliminary data from these theses have previously been published as an abstract in the Proceedings of the ACVS Surgery Summit 2023, 12–14 October, Louisville, Kentucky. Vet Surg 52: O1-O71. https://doi.org/10.1111/vsu.14012. The study was part of a doctoral thesis, and a summary of the results has been published in the compilation part of the thesis https://res.slu.se/id/publ/132963.
