# Supplementary material for: Equine castration in Sweden: a retrospective study of postoperative complications across field and hospital practices
Source: Acta Vet Scand. 2026 Nov 13;68:54. doi: 10.1186/s13028-026-00889-y (PMC13769181; doi:10.1186/s13028-026-00889-y)
Supplement: Supplementary file 1 [file 13028_2026_889_MOESM1_ESM.docx]

**Additional file 1**

Results of the univariable analysis in the full study population, in the Field group and in the Hospital group.

**Table 1.** Associations between selected variables and complication in the full study population.

| **Variable (n)** | **df** | **χ^2^** | **P-value** |
| --- | --- | --- | --- |
| Age category (987^#^) | 2 | 2.853 | 0.2401 |
| Breed (1075) | 5 | 45.349 | <0.0001 |
| Group (1075) | 1 | 71.828 | <0.0001 |

^#^ Missing values 88 (all within the Field group)
P-values obtained using the likelihood ratio test

**Table 2.** Associations between selected variables and surgical site infection in the full study population.

| **Variable (n)** | **df** | **χ^2^** | **P-value** |
| --- | --- | --- | --- |
| Age category (987^#^) | 2 | 2.683 | 0.2614 |
| Breed (1075) | 5 | 62.614 | <0.0001 |
| Group (1075) | 1 | 101.785 | <0.0001 |

^#^ Missing values 88 (all within the Field group).
P-values obtained using the likelihood ratio test.

**Table 3**. Associations between explanatory variables and complication in the Field group**.**

| **Variable (n)** | **DF** | **χ^2^** | **P-value** |
| --- | --- | --- | --- |
| Age category (433^#^) | 2 | 2.668 | 0.2634 |
| Breed (521) | 5 | 7.803 | 0.1675 |
| Season (521) | 3 | 0.659 | 0.8828 |
| Antimicrobial prophylaxis (521) | 2 | 8.194 | 0.0166 |
| NSAID perioperative (521) | 1 | 0.042 | 0.8380 |
| NSAID postoperative (521) | 1 | 10.713 | 0.0011 |
| Technique (230) | 1 | 0.000 | 0.9974 |

^#^ Missing values 88.
P-values obtained using the likelihood ratio test.

**Table 4.** Associations between explanatory variables and surgical site infection in the Field group**.**

| **Variable (n)** | **DF** | **χ^2^** | **P-value** |
| --- | --- | --- | --- |
| Age Category (433^#^) | 2 | 0.554 | 0.7581 |
| Breed (521) | 5 | 10.305 | 0.0671 |
| Season (521) | 3 | 1.311 | 0.7266 |
| Antimicrobial prophylaxis (521) | 2 | 10.784 | 0.0046 |
| NSAID perioperative (521) | 1 | 0.324 | 0.5692 |
| NSAID postoperative (521) | 1 | 10.554 | 0.0012 |
| Technique (230) | 1 | 0.051 | 0.8209 |

^#^ Missing values 88.
P-values obtained using the likelihood ratio test.

**Table 5**. Associations between explanatory variables and complication in the Hospital group**.**

| **Variable (n)** | **DF** | **χ^2^** | **P-value** |
| --- | --- | --- | --- |
| Age Category (554) | 2 | 9.574 | 0.0083 |
| Breed (554)^#^ | 5 | 4.989 | 0.4172 |
| Season (554) | 3 | 2.757 | 0.4306 |
| Antibiotics postoperative (554) | 1 | 0.170 | 0.6799 |
| NSAID postoperative (554) | 1 | 0.011 | 0.9164 |
| Technique (554) | 2 | 1.829 | 0.1763 |
| Primary suturing (554) | 1 | 0.013 | 0.9079 |
| Hospital (554) | 2 | 6.431 | 0.0401 |

^#^Number of complications were less than 5 in all breeds except Standardbred trotters and warmblood horses.
P-values obtained using the likelihood ratio test.

**Table 6.** Associations between explanatory variables and surgical site infection in the Hospital group**.**

| **Variable (n)** | **DF** | **χ^2^** | **P-value** |
| --- | --- | --- | --- |
| Age Category (554) | 2 | 4.787 | 0.0913 |
| Breed (554) ^#^ | 5 | 7.569 | 0.1816 |
| Season (554) | 3 | 9.444 | 0.0239 |
| Antibiotics postoperative (554) | 1 | 0.232 | 0.6301 |
| NSAID postoperative (554) | 1 | 1.680 | 0.1950 |
| Technique (554) | 2 | 0.047 | 0.8277 |
| Primary suturing (554) | 1 | 0.039 | 0.8436 |
| Hospital (554) | 2 | 0.309 | 0.8566 |

^#^Number of surgical site infections were less than 5 in all breeds except Standardbred trotters and warmblood horses.
P-values obtained using the likelihood ratio test.

**Table 2.** Associations between explanatory variables and surgical site infection in the Hospital group**.**

| **Variable (n)** | **DF** | **χ^2^** | **P-value** |
| --- | --- | --- | --- |
| **Hospital** |  |  |  |
| Age Category (554) | 2 | 4.787 | 0.0913 |
| Breed (554) | 5 | 7.569 | 0.1816 |
| Season (554) | 3 | 9.444 | 0.0239 |
| Antibiotics postoperative (554) | 1 | 0.232 | 0.6301 |
| NSAID postoperative (554) | 1 | 1.680 | 0.1950 |
| Technique (554) | 2 | 0.047 | 0.8277 |
| Primary suturing (554) | 1 | 0.039 | 0.8436 |
| Hospital (554) | 2 | 0.309 | 0.8566 |

P-values obtained using the likelihood ratio test.
