# Supplementary material for: Equine castration in Sweden: a retrospective study of postoperative complications across field and hospital practices
Source: Acta Vet Scand. 2026 Nov 13;68:54. doi: 10.1186/s13028-026-00889-y (PMC13769181; doi:10.1186/s13028-026-00889-y)
Supplement: Supplementary file 2 [file 13028_2026_889_MOESM2_ESM.docx]

**Additional file 2**

**Table 1.** Odds ratio of complication in the full study population after multivariable logistic regression.

| **Variable** | **Category** | **OR (95% CI)** | **P-value** |
| --- | --- | --- | --- |
| Group (n) | Hospital (554) *ref.* | 1.0 |  |
|  | Field (521) | **4.5 (3.18–6.36)** | **<0.0001** |
| Age (n) ^#^ | ≤ 2 (567) *ref.* | 1.0 |  |
|  | 3-4 (311) | 1.1 (0.78­–1.60) | 0.5457 |
|  | ≥ 5 (109) | **2.5 (1.50–4.24)** | **0.0006** |

^#^ Missing values 88
Significant values in bold
Abbreviations: *ref*; reference

**Table 2.** Odds ratio of surgical site infection in the full study population after multivariable logistic regression.

| **Variable** | **Category** | **OR (95% CI)** | **P-value** |
| --- | --- | --- | --- |
| Group (n) | Hospital (554) *ref.* | 1.0 |  |
|  | Field (521) | **5.9 (3.43–9.99)** | **<0.001** |
| Breed (n) | Cold-blooded horses (82) *ref.* | 1.0 |  |
|  | Icelandic horses (76) | 0.6 (0.28­–1.23) | 0.155 |
|  | Other (158) | **0.4 (0.20–0.69)** | **0.002** |
|  | Pony (119) | **0.5 (0.26–0.92)** | **0.026** |
|  | STB (331) | **0.3 (0.16–0.74)** | **0.006** |
|  | WBL (309) | **0.4 (0.25–0.80)** | **0.007** |

Significant values in bold
Abbreviations: *ref*; reference, STB; Standardbred trotter, WBL; warmblood horse
