# Supplementary material for: Equine castration in Sweden: a retrospective study of postoperative complications across field and hospital practices
Source: Acta Vet Scand. 2026 Nov 13;68:54. doi: 10.1186/s13028-026-00889-y (PMC13769181; doi:10.1186/s13028-026-00889-y)
Supplement: Supplementary file 3 [file 13028_2026_889_MOESM3_ESM.docx]

**Additional file 3**

**Table 1.** Odds ratio of complication in the Field group after multivariable logistic regression (N=521).

| **Variable** | **Category** | **OR (95% CI)** | **P-value** |
| --- | --- | --- | --- |
| Antimicrobial Ppx. (n) | Perioperative (149) *ref.* | 1.0 |  |
|  | No (286) | **1.5 (1.01–2.36)** | **0.0469** |
|  | Extended (86) | 0.7 (0.40–1.34) | 0.3183 |
| NSAID postoperative (n) | No (145) *ref.* | 1.0 |  |
|  | Yes (376) | **2.1 (1.36–3.27)** | **0.0007** |

Significant values in bold
Abbreviations: Ppx; prophylaxis, *ref*; reference

**Table 2.** Odds ratio of surgical site infection in the Field group after multivariable logistic regression (N=521).

| **Variable** | **Category** | **OR (95% CI)** | **P-value** |
| --- | --- | --- | --- |
| Breed | Cold-blooded horses (n=71) *ref.* | 1.0 |  |
|  | Icelandic horses (46) | 0.6 (0.24–1.29) | 0.172 |
|  | Other (127) | **0.4 (0.19–0.70)** | **0.003** |
|  | Ponies (105) | **0.5 (0.26–0.98)** | **0.043** |
|  | STB (28) | 0.4 (0.13–1.02) | 0.055 |
|  | WBL (144) | **0.4 (0.21–0.75)** | **0.004** |
| Antimicrobial Ppx. (n) | Perioperative (149) *ref.* | 1.0 |  |
|  | No (286) | **1.7 (1.08–2.82)** | **0.023** |
|  | Extended (86) | 0.7 (0.33–1.39) | 0.282 |
| Postoperative NSAID (n) | No (145) *ref.* | 1.0 |  |
|  | Yes (376) | **2.5 (1.47–4.11)** | **0.001** |

Significant values in bold
Abbreviations: Ppx; prophylaxis, *ref*; reference, STB; Standardbred trotter, WBL; warmblood horse
