# Supplementary material for: Equine castration in Sweden: a retrospective study of postoperative complications across field and hospital practices
Source: Acta Vet Scand. 2026 Nov 13;68:54. doi: 10.1186/s13028-026-00889-y (PMC13769181; doi:10.1186/s13028-026-00889-y)
Supplement: Supplementary file 4 [file 13028_2026_889_MOESM4_ESM.docx]

**Additional file 4**

**Table 1.** Odds ratio of complication in the Hospital group after multivariable logistic regression (N=554).

| **Variable** | **Category** | **OR (95% CI)** | **P-value** |
| --- | --- | --- | --- |
| Age (n) | ≥ 5 (95) *ref.* | 1.0 |  |
|  | ≤ 2 (252) | **0.3 (0.17-0.63)** | **0.0009** |
|  | 3–4 (207) | **0.4 (0.23-0.83)** | **0.0118** |
| Hospital (n) | C (60) *ref.* | 1.0 |  |
|  | A (288) | **3.4 (1.15-14.65)** | **0.0245** |
|  | B (206) | **4.5 (1.51-19.29)** | **0.0049** |

Abbreviations: *ref*; reference

**Table 2.** Odds ratio of surgical site infection in the Hospital group after multivariable logistic regression (N=554).

| **Variable** | **Category** | **OR (95% CI)** | **P-value** |
| --- | --- | --- | --- |
| Season (n) | Winter (118) *ref.* | 1.0 |  |
|  | Spring (187) | **6 (1.1–109.8)** | **0.0377** |
|  | Summer (98) | 4 (0.46–75.3) | 0.2239 |
|  | Autumn (151) | **10 (1.9–185.3)** | **0.0030** |

Significant values in bold
Abbreviations: *ref*; reference
